# Identification of Potential Gene Targets for Suppressing Oviposition in *Holotrichia parallela* Using Comparative Transcriptome Analysis

**DOI:** 10.3390/ijms241713138

**Published:** 2023-08-24

**Authors:** Zhongjun Gong, Jing Zhang, Yanmin Li, Huiling Li, Ziqi Zhang, Yifan Qin, Yueli Jiang, Yun Duan, Tong Li, Jin Miao, Yuqing Wu

**Affiliations:** 1 Institute of Plant Protection, Henan Academy of Agricultural Sciences, Key Laboratory of Crop Pest Control of Henan Province, Key Laboratory of Crop Integrated Pest Management of the Southern of North China, Ministry of Agriculture of the People’s Republic of China, Zhengzhou 450002, China; gongzj_2@hotmail.com (Z.G.);; 2 Institute of Horticulture, Henan Academy of Agricultural Sciences, Zhengzhou 450002, China; 3 Institute of Plant Protection, Luoyang Academy of Agricultural and Forestry Sciences, Luoyang 471027, China

**Keywords:** *Holotrichia parallela*, plant–insect interaction, comparative transcriptome, follicle cell protein 3c, oviposition

## Abstract

*Holotrichia parallela* is an important plant pest. Comparative feeding experiments showed that the egg production, oviposition duration and survival rate of *H. parallela* beetles were significantly higher when they fed on elm leaves than when they fed on willow or purpus privet leaves. RNA sequencing was used to determine transcriptomic changes associated with oviposition. Comparative transcriptome analysis revealed that the beetles that fed on elm and willow had a total of 171 genes with differential expression. When the beetles fed on elm and purpus privet, 3568 genes had differential expression. The vitellogenesis, ovarian serine protease, odorant-binding proteins, acyl-CoA synthetase and follicle cell proteins were commonly upregulated genes in elm-fed beetles compared with those fed on willow/purpus privet leaves. The involvement of the follicle cell protein 3C gene in the regulation of oviposition was confirmed using RNA interference. The results provide insights into the molecular mechanisms underlying oviposition in *H. parallela* feeding on different host plants. This study also describes a method for identifying potentially effective genes for pest control.

## 1. Introduction

The dark black chafer *Holotrichia parallela* Motschulsky (Coleoptera: Scarabaeoidea) is a pest of agricultural and horticultural crops in China [1]. Economic losses result from adults eating leaves and fruits and larvae feeding on roots and stems [2]. Conventional chemical treatments such as seed coating or soil drenching result in soil pollution and diminish the quality of peanut products [3]. Therefore, it is important to identify molecular targets involved in the oviposition of *H. parallela* to develop more effective, environmentally safe control techniques.

The reproductive rate is fundamental for maintaining insect populations [4]. A high reproductive capacity can increase offspring numbers and lead to pest outbreaks [5]. Nutrients are critical for successful insect reproduction [6,7]. The nutritive value and metabolite composition of host plant species vary and influence insect development and reproduction [8,9,10]. The effects of feeding adults of *H. parallela* with 17 different plants, such as elm, willow and purpus privet leaves, have been determined. There were significant differences in fecundity among the different host plant treatments [11]. Another study found that *H. parallela* adults fed with elm leaves had the highest fecundity [12].

Transcriptome sequencing is an accurate and efficient deep sequencing technology that serves as a powerful analysis tool, especially for species that lack reference genomic information [13]. Transcriptome sequencing revealed 89 potential chemosensory receptors in the antennal transcriptome of *H. parallela* [14]. Additionally, Cry8Ea-binding proteins have been found in the midgut tissue of *H. parallela* [15]. Transcriptome sequencing has also generated a resource for *H. parallela* larvae and has helped in the identification of immune defense-related differentially expressed genes (DEGs) in response to nematode infection [16]. Analysis of *H. parallela* larvae transcriptomics in response to EPN-Bt infection identified important antioxidant and detoxifying enzyme genes [17]. However, there is limited information about the changes in gene expression associated with oviposition, as well as the primary signal transduction pathways in *H. parallela*.

Our initial study objective was to confirm the current understanding of the oviposition behavior of *H. parallela* when they were provided with leaves from elm (*Ulmus pumila* L.), willow (*Salix babylonica* L.), and purpus privet (*Ligustrum quihoui* Carr.). Our main hypothesis was that the variation in oviposition-related genes in the ovaries of *H. parallela* can be influenced by different diets. To study the variations in oviposition and the mechanisms underlying the response of *H. parallela* to different host plants, we sequenced the gonadal transcriptomes of *H. parallela* fed on these three host plants. The genes involved in oviposition as well as major signal transduction pathways were identified. The function of a crucial gene, follicle cell protein 3C (Fcp3C), was examined using RNA interference (RNAi). The findings provide a deeper understanding of the molecular mechanisms involved in *H. parallela* oviposition. These results offer potential strategies useful for pest management.

## 2. Results

### 2.1. Effects of Different Diets on Reproduction

Differences in survival rate, fecundity and oviposition duration were observed in the three diet treatments. The mean fecundity (eggs) of females reared on elm was the highest (67.4), followed by that of females fed on willow (21.1). The fecundity of *H. parallela* reared on purpus privet was the lowest (8.6) (Table 1).

Oviposition duration was also significantly different among treatments. *H. parallela* reared on elm leaves had a 59.3 d oviposition period. In contrast, beetles reared on willow and purpus privet leaves had oviposition durations of 46.7 and 39.3 d, respectively. The highest survival rate (90.0%) was observed when beetles were reared on elm leaves (Table 1).

### 2.2. Sequencing and Analysis of Reproductive Tissues at the Transcriptome Level

Adult ovary and testis cDNA libraries were prepared from *H. parallela* reared on three different diet treatments, which were the elm (OU group), willow (OS group) and purpus privet (OL group). The tissues underwent Illumina sequencing. A total of 870.9 million raw reads were generated through Illumina paired-end sequencing. Following cleaning and quality control steps, 834.4 million clean reads (115.1Gb) were obtained, with an error rate of <0.02%. The bases in the sequencing had a Q30 percentage of >89%, indicating high reliability (Table S2). The assembly results showed that 243,432 unigenes were generated with a 556 bp mean length and a 708 bp N50 value (Table S3). Overall, 60,190 (24.72%), 29,278 (12.02%), 18,007 (7.39%), 37,023 (15.2%), 42,094 (17.29%), 16,898 (6.94%) and 43,133 (17.71%) unigenes were annotated in the databases of Nr, Nt, Kyoto Encyclopedia of Genes and Genomes (KEGG), SwissProt, Pfam, KOG and GO, respectively (Table S3). In the NR database, the 60,181 annotated unigenes matched known sequences from different insect species. The *H. parallela* sequences showed 25.5% matches with *Tribolium castaneum* sequences, followed by *Lasius niger* (5.3%), *Harpegnathos saltator* (4.1%) and *Bombyx mori* (3.7%) (Figure S1).

**Figure 1:**
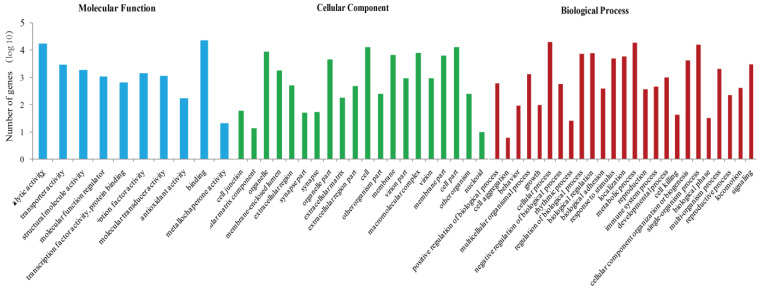
Gene ontology (GO) assignment for the *Holotrichia parallela* unigenes.

In the GO annotation, total unigenes were classified into 55 functional groups across three main categories: biological process, cellular component and molecular function. Each category had predominant terms, with “cellular process”, “cell” and “binding” being the most common terms in their respective categories (Figure 1).

Out of the 26 KOG categories, the largest group was represented by the cluster for “General function prediction only” at 14.0%. This was followed by “Posttranslational modification, protein turnover, chaperones” at 13.5%, “Translation, ribosomal structure, and biogenesis” at 11.7% and “Signal transduction mechanisms” at 9.7% (Figure S2).

**Figure 2:**
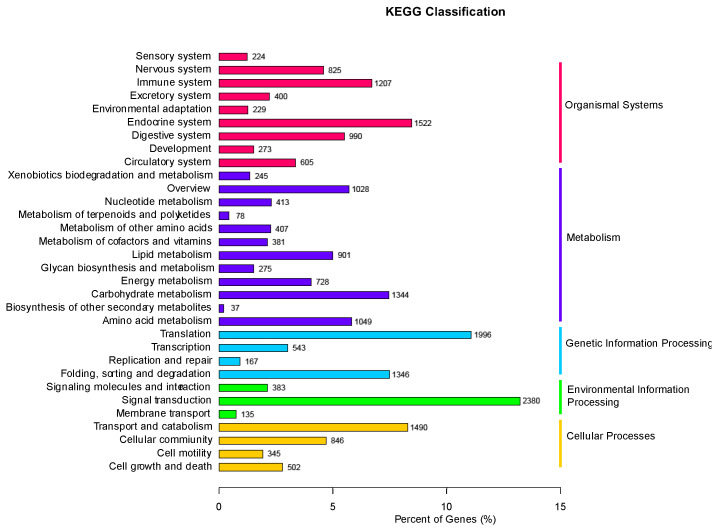
Classification of DEGs according to the pathway type in the KEGG database.

KEGG analysis was used to classify the annotated genes into different pathway functional categories. The most representative pathways included “Signal transduction”, “Translation”, and “Endocrine system” (Figure 2).

### 2.3. Differentially Expressed Genes in Response to Different Hosts

The DEGs among different host plants were identified using DEGseq. In total, 171 genes were discovered, with 81 genes showing downregulation and 90 genes showing upregulation in the OS group compared with the OU group. Additionally, a total of 3568 DEGs were identified, with 2827 genes showing downregulation and 741 genes showing upregulation in the OL group compared with the OU group. Among the downregulated genes, 65 were downregulated in both treatments (Table S4).

To study the functions of DEGs, we conducted KEGG pathway analyses. KEGG pathway analysis revealed that, of the 85 pathways analyzed, 24 pathways showed significant enrichment (*p* < 0.05) among the DEGs between OS and OU. These enriched pathways included several pathways that play a vital role in ovary development, such as oxidative phosphorylation (ko00190) and cell adhesion molecules (ko04514). The top 20 most significantly enriched metabolic pathways are listed in Table S5.

KEGG pathway analysis revealed that among the DEGs between OL and OU, a total of 285 pathways were involved. Out of these, 35 pathways showed significant enrichment (*p* < 0.05). These enriched pathways included several pathways associated with ovary development, such as oxidative phosphorylation (ko00190), pyrimidine metabolism (ko00240) and protein processing in the endoplasmic reticulum (ko04141). The top 20 most significantly enriched metabolic pathways are listed in Table S6.

### 2.4. Oviposition-Related Genes

**Figure 3:**
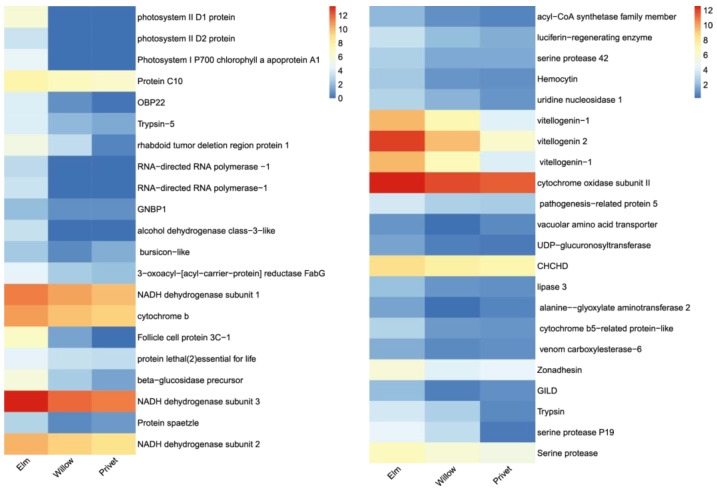
Expression heat map of reproduction or development-related unigenes in the ovaries of *Holotrichia parallela* that were fed on elm, willow or privet leaves. The expression level of each unigene in different treatments is represented by horizontal lines. To analyze the data, the mean log2 (FPKM + 1) value of each gene in each group was normalized.

The process of ovarian development plays a critical role in insect reproduction. Hence, the identification of genes associated with oviposition is important because it provides a better understanding of the molecular mechanisms underlying insect reproduction. We found that the expression levels of many genes involved in development were significantly altered (*p* < 0.05), including vitellogenesis, ovarian serine protease (Osp), odorant-binding proteins (OBPs), acyl-CoA synthetase and follicle cell proteins (Table S7). A heat map was created to visualize and analyze the variations between the different treatments (Figure 3).

### 2.5. Phylogenetic Analysis

**Figure 4:**
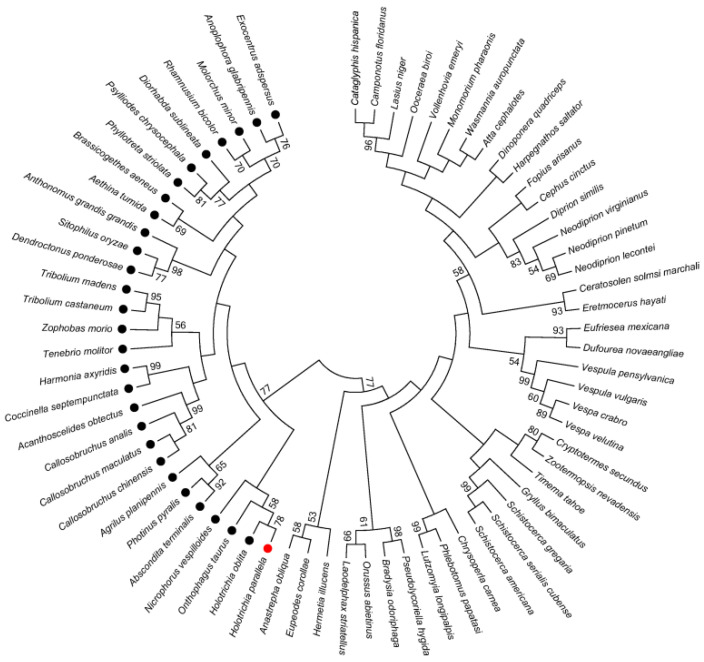
Phylogenetic relationships of 70 Fcp3C-like genes in insect species. MEGA6 was used to construct the phylogenetic tree through maximum likelihood analyses with 1000 bootstrap replications. Bootstrap scores higher than 50% are displayed on the nodes. The dots on the tree indicate Fcp3C protein from beetles.

To investigate the evolutionary conservation of the Fcp3C genes, homologous sequences were selected from 70 insect species. The species included representatives from Lepidoptera, Coleoptera, Hemiptera, Hymenoptera, Orthoptera, Blattodea, Phasmatoptera and Diptera. The findings indicated a high level of conservation in the Fcp3C protein sequence (Figure 4).

### 2.6. Gene Expression Analysis

**Figure 5:**
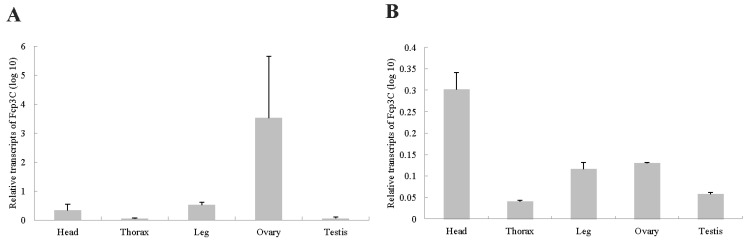
Expression of Fcp3C (**A**) and FoxL2 (**B**) in different tissues of adult *Holotrichia parallela* using qPCR. Data are presented as means ± SE of three independent biological replicates.

The expression patterns of Fcp3C and forkhead box L2 (FoxL2) in various tissues, including the head, thorax, leg, testis and ovary, were analyzed using qPCR. The ovary exhibited the highest expression level of Fcp3C, while the testis and other tissues showed relatively low levels. The expression level of FoxL2 was higher in the head than in the ovary (Figure 5).

### 2.7. Effect of Fcp3C on Oviposition

To determine the role of Fcp3C in the oogenesis of adult females of *H. parallela*, we performed RNAi knockdown experiments. We specifically examined the influence of Fcp3C knockdown on the daily egg-laying count and hatchability. The efficiency of knockdown was assessed through qPCR on the 7th day post-feeding.

**Figure 6:**
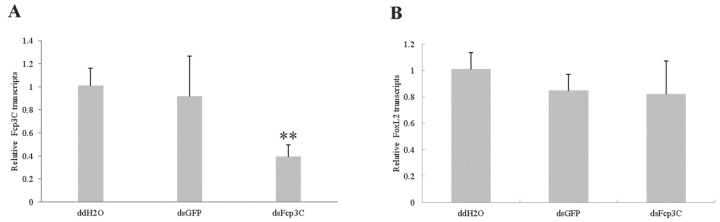
RNAi efficiency of Fcp3C (**A**) and FoxL2 (**B**) in the ovary of adult females on the 7th day post-feeding dsFcp3C. dsRNA was quantified through qPCR. Data are presented as means ± SE of three independent biological replicates. ** *p* < 0.01, which was determined by Duncan’s multiple range test, indicates a significant difference compared with the control groups of ddH_2_O and dsGFP.

The RNAi analysis showed that expression of the Fcp3C genes in the ovary under the dsFcp3C treatment was significantly suppressed compared with the levels in the ddH_2_O and dsGFP control groups (Figure 6). Fcp3C expression declined by an average of 61.2%. Feeding dsGFP did not affect Fcp3C expression. The FoxL2 gene is related to ovarian development and was also tested. Feeding dsFcp3C did not affect FoxL2 expression levels.

**Figure 7:**
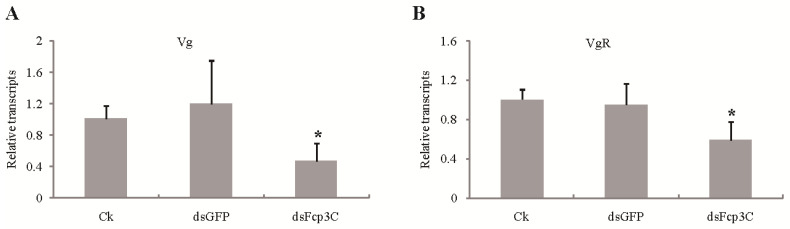
Relative expression levels of Vg (**A**) and VgR (**B**) in 10 female adults fed on dsFcp3C using qPCR. Data are presented as means ± SE of three independent biological replicates. * *p* < 0.05, which was determined by Duncan’s multiple range test, indicates a difference compared with the control groups of ddH_2_O and dsGFP.

The vital roles of vitellogenin (Vg) and vitellogenin receptor (VgR) in insect vitellogenesis have been established. To expand our understanding, we conducted investigations on the effects of Fcp3C RNAi on the expression of oogenesis-related genes, Vg and VgR, in *H. parallela*. The knockdown of Fcp3C significantly decreased the expression of Vtg in ovarian development. Similar to Vtg, the knockdown of Fcp3C also resulted in a significant decrease in the expression of vitellogenin receptor (VgR) in the ovary. The expression levels of Vg and VgR were decreased by approximately 53.4% and 41.1%, respectively (Figure 7).

**Figure 8:**
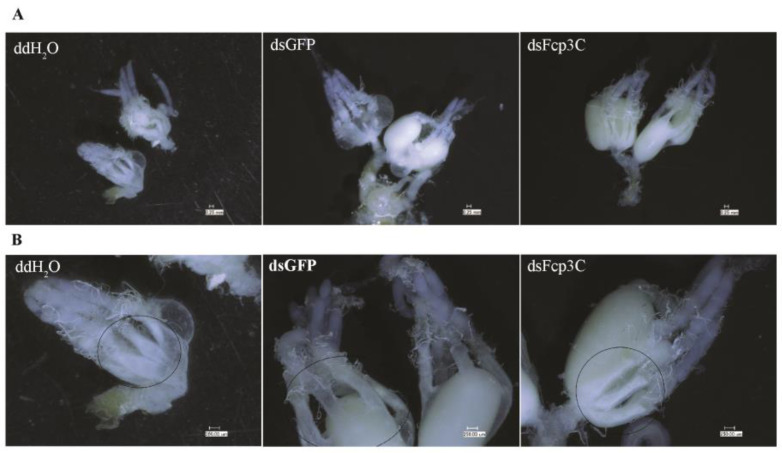
Effect of RNAi on the ovaries in the ddH_2_O, dsGFP (control), and dsFcp3C groups (**A**) and enlarged view (**B**). There was no significant difference in oocyte morphology among the three groups. On the 11th day, all ovarian samples dissected from the dsFcp3C treatment females (n = 10) contained oocytes, while the developed oocytes in more than 50% of the ovaries of the adult females treated with ddH_2_O and dsGFP were oviposited. The photos were taken with a VHX-7000 digital microscope (KEYENCE, Mechelen, Belgium).

Dissection of the female adult ovaries showed that oocytes in the dsFcp3C group had normal morphology on the 11th day. On that day, all ovarian samples dissected from the dsFcp3C treatment females (n = 10) contained oocytes, while more than 50% of the ovaries in adult females treated with ddH_2_O and dsGFP oviposited all of their eggs. In the circular area of the dsFcp3C treatment group, the internal membrane was not highly permeable compared with the internal membrane in the other groups (Figure 8).

**Figure 9:**
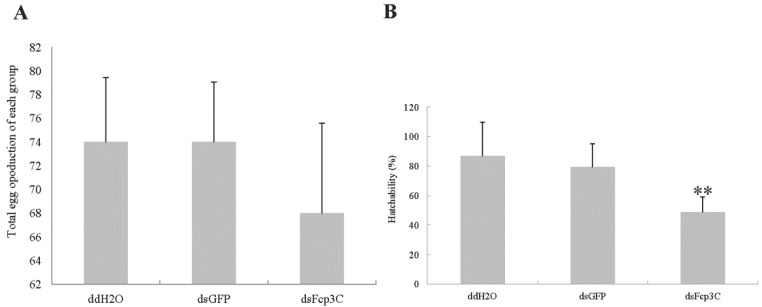
Egg production (**A**) and hatchability (**B**) in 30 female adults fed with dsFcp3C. Data are presented as means ± SE of six independent biological replicates. ** indicates an extremely significant difference determined by Duncan’s multiple range test.

The knockdown of Fcp3C suppressed the hatching rate but did not affect the oviposition number (Figure 9). The eggs of adult females treated with dsFcp3C had a 43.8% decrease in offspring compared with the number of offspring produced by the ddH_2_O controls.

## 3. Discussion

We explored the influence of diet on the oviposition of *H. parallela*. We also identified many DEGs in *H. parallela* fed on three different host plants using the comparative transcriptome method. Many genes had lower expression in the ovarian tissues of *H. parallela* that fed on willow or purpus privet leaves than in the ovarian tissues of beetles that fed on elm leaves. This result suggests that these genes have developmental or reproductive functions.

Transcriptome sequencing is a useful tool for global gene expression analysis [13,18]. However, when only two groups are compared, hundreds of DEGs are often identified [19,20,21]. It is challenging to quickly screen and identify the most effective genes among the hundreds of DEGs. Further analysis, such as functional enrichment analysis or pathway analysis, may be required to prioritize and identify key genes that are biologically relevant and potentially involved in the observed differences between the two groups. We used three treatments in this study. One was the preferred host plant (elm), and the other two treatments were alternative host plants (willow and privet). Only 65 downregulated genes were found, which narrowed the scope of screening for effective genes.

Differences in nutritional value among the host plants may affect the ovaries [22] and affect development and reproduction. Many studies have evaluated the biological parameters of *H. parallela* reared on different host plants [11,12,23]. We demonstrated that *H. parallela* feeding on elm had a higher adult survival rate, fecundity and oviposition duration than beetles fed on willow and privet. This indicates that elm is a more suitable host plant for the growth and survival of *H. parallela*. To ensure safety and feasibility in integrated pest management programs, it is important to understand the molecular mechanisms underlying oviposition regulation in *H. parallela* by host plants and to develop environment-friendly targets for pest control. Previous research has studied the mechanism of oviposition-related genes and has provided insights into the interactions among the genes involved in insect reproduction [24]. However, there is less research on the influence of host plants on the gonads of insects and underlying molecular mechanisms. We conducted a comparative transcriptome analysis on the gonads of *H. parallela* that were fed on elm, willow or privet leaves and identified many DEGs.

The regulation of vitellogenesis in insects involves endocrine hormones, and the role of nutrition is also significant in most female insects [25]. Insufficient levels of one or more nutrients can affect the transcriptional synthesis of Vg. Vg is an essential biological marker for ovarian development. It is a pivotal gene that regulates hormone signal transduction and egg yolk formation and provides protection against oxidative stress [24,26]. Females of *Octodontanipae*, when fed on their preferred host *Phoenix canariensis*, exhibited significantly higher expression levels of Vg and higher egg hatchability compared with females fed on *Phoenix roebelenii* [27]. We found that the Vg gene was significantly downregulated in OS and OL *H. parallela* females compared with those in the OU treatment. This indicates that the nutritional content in the OU treatment (elm leaves) was more suitable for vitellogenesis. The knockdown of HparFcp3C resulted in a significant decrease in the expression of HparVg and HparVgR in the ovary, indicating the role of HparFcp3C in regulating the transport of HparVg and HparVgR.

In addition to the genes Vg and Fcp3C, the annotation results based on DEGs also revealed the identification of one OBP that is a candidate reproductive protein in insects. The expression levels of the unigenes annotated to OBP were lower in OS and OL groups than in the OU group. The roles of OBPs have been characterized in terms of their involvement in detecting plant volatiles, sex pheromones, and oviposition repellents, as well as other unknown roles [28,29,30,31].

Gram-negative binding protein, also known as β-1,3-glucan-binding protein, is a pattern recognition protein involved in insect immunity against entomopathogens [32,33,34]. The role of Acyl-CoA synthetase family member 2 in insect reproduction is linked to its function in producing the intracellular acyl-CoA pool. This pool is exclusively utilized for β-oxidation in the fat body, and it influences reproductive processes. [35]. The two genes were expressed at lower levels in the OS and OL groupsthan in the OU group.

The Osp gene is responsible for encoding a serine protease family member involved in the development of ovaries and eggs [36,37]. The deletion of Osp genes in *B. mori* and *Spodoptera litura* causes infertility in females [36,37]. The proteins encoded by the Osp genes exhibit a high level of conservation in Lepidoptera and Coleoptera [36]. This gene was also downregulated in the OS and OL groups compared with the OU group. The Osp gene may have a similar function of reducing egg production in *H. parallela*. The Osp gene shows potential as a molecular target for genetic-based pest management approaches in many insect species.

Fcp3C was first identified in *Drosophila melanogaster* and plays a crucial role in chorion formation [38,39,40]. In *Diploptera punctata*, the expression patterns also indicate that Fcp3C is involved in the process of chorion formation [40]. Depletion of BgWindei through RNAi disrupted chorion formation and was accompanied by a significant decrease in the expression of Fcp3C in *Blattella germanica* [41]. In *H. parallela*, the expression of Fcp3C was significantly reduced in the OS and OL groups compared with the OU group. The knockdown of Fcp3C resulted in the suppression of Vg and VgR expression in *H. parallela*, indicating the significant involvement of Fcp3C in reproductive processes. The knockdown of Fcp3C suppressed the hatching rate but did not affect the number of eggs laid. In contrast, the knockdown of Fcp3C in *Nilaparvata lugens* resulted in an increased number of eggs. Species-specific variations may lead to divergent responses following the knockdown of Fcp3C.No significant variation was observed in the expression of the FoxL2 gene among the three *H. parallela* groups. Fcp3C is a downstream gene of FoxL2 in *N. lugens* [38]. Therefore, based on differences in gene expression, it appears that the genetic network involving Fcp3C and FoxL2 in *H. parallela* differs from that of *N. lugens*. Further research is required to uncover more detailed mechanisms. Aside from investigating novel genes, it is crucial to examine the relationships between upstream and downstream genes and establish a comprehensive gene repository of oviposition-related genes. This would allow for a more holistic understanding of the genetic regulation of oviposition and facilitate further research in this field. An important future direction for the study of insect oviposition behavior is the utilization of RNAi technology to validate the functions of identified genes in female oviposition behavior.

The use of RNAi to silence gene expression has proven to be highly effective and systemic in Coleoptera species [42,43,44,45,46]. Our data demonstrate the feasibility of using bacteria as a delivery system for inducing RNAi and efficiently controlling *H. parallela*. The production of dsRNA by bacteria is cost-effective and easily scalable for large-scale production [46].Before considering the implementation of feeding RNAi as a pest control method, it is important to address several potential concerns in future studies. These concerns include the potential effects on non-target species and the potential for resistance development [45,47]. Therefore, further studies could investigate the optimization of the delivery system to enhance the efficiency and specificity of RNAi induction. Additionally, the potential non-target effects and species specificity of the delivered dsRNA require evaluation.

## 4. Materials and Methods

### 4.1. Tissue Sampling

*Holotrichia parallela* adults were originally collected from the Luoyang Academy of Agriculture and Forestry Sciences (Luoyang City, Henan Province, China, 34°38′ N, 112°29′ E) and the Henan Research and Development Center for Modern Agriculture (Yuanyang County, Henan Province, China, 35°00′ N, 113°40′ E). To reduce the potential influence of age-related variation, we used a sampling strategy focused on an early-life stage of the beetle. The beetles were separated by gender and placed in containers with soil and fresh leaves. They were reared under controlled conditions of 25 ± 1 °C, 70% relative humidity, and a 12L:12D photoperiod. During the rearing period, the fresh leaves of elm (*Ulmus pumila* L.), willow (*Salix babylonica* L.), and purpus privet (*Ligustrum quihoui* Carr.) were provided as food and were replaced with fresh leaves each day. For each of the different treatments, we performed three replicates with 20 males and 20 females in plastic boxes. The biology parameters (adult longevity, survival rate, fecundity and oviposition duration) of adult development were recorded every three days.

### 4.2. RNA Extraction and Sequencing

Different transcriptome profiles were generated from the ovaries and testes of *H. parallela* beetles in all feeding treatment groups, except for the tastes from the purpus privet treatment group. In total, 15 cDNA libraries were prepared. A total of 3 µg of RNA per sample was used as input material for the RNA sample preparation. To ensure the quality of the RNA, degradation and contamination were monitored on 1% agarose gels. Additionally, RNA purity was assessed using a NanoPhotometer spectrophotometer (IMPLEN, Westlake Village, CA, USA). The concentration of RNA was measured with the Qubit RNA Assay Kit using the Qubit 2.0 Fluorometer (Life Technologies, Carlsbad, CA, USA). Furthermore, RNA integrity was evaluated using the RNA Nano 6000 Assay Kit with the Agilent Bioanalyzer 2100 (Agilent Technologies, Santa Clara, CA, USA).

Sequencing libraries were generated using the NEBNext^®^ Ultra™ RNA Library Prep Kit for Illumina (NEB, Beverly, MA, USA), following the manufacturer’s instructions. Index codes were added to attribute sequences to each sample. Briefly, mRNA was purified from total RNA using poly-T oligo-attached magnetic beads. Fragmentation was performed using divalent cations under elevated temperature in NEBNext First Strand Synthesis Reaction Buffer (5×). The synthesis of first-strand cDNA was achieved using a random hexamer primer and M-MuLV Reverse Transcriptase (RNase H-). Subsequently, second-strand cDNA synthesis was performed using DNA Polymerase I and RNase H. The remaining overhangs were then converted into blunt ends through the activities of exo-nucleases and polymerases. Following the adenylation of the 3′ ends of DNA fragments, NEBNext Adaptor with a hairpin loop structure was ligated to facilitate hybridization preparation. To isolate cDNA fragments ranging from 150 to 200 bp in length, the library fragments were purified using the AMPure XP system (Beckman Coulter, Brea, CA, USA). Subsequently, 3 µL of USER Enzyme (NEB) was added to the size-selected, adaptor-ligated cDNA and incubated at 37 °C for 15 min, followed by a 5 min incubation at 95 °C to inactivate the enzyme. PCR amplification was then performed using Phusion High-Fidelity DNA polymerase, Universal PCR primers and Index (X) Primer. Finally, the PCR products were purified using the AMPure XP system, and the quality of the library was evaluated using the Agilent Bioanalyzer 2100 system. The index-coded samples were clustered on a cBot Cluster Generation System using the TruSeq PE Cluster Kit v3-cBot-HS (Illumina), following the manufacturer’s instructions.

After cluster generation, the library preparations were sequenced on an Illumina Hiseq 2500 platform, and paired-end reads were generated. RNA sequencing was performed by Novogene Bioinformatics Technology Co., Ltd. (Beijing, China) on the Illumina Hiseq 2500 platform.

### 4.3. Transcriptome Assembly and Analysis

To generate a reference transcriptome for *H. parallela*, a total of 15 cDNA libraries were combined and subjected to de novo assembly. Trinity [48] was used to assemble the transcriptome based on the reads, with the default settings for all parameters except for the min_kmer_cov (accessed on 31 October 2017), which was set to 2. Functional annotations of all assembled unigenes were conducted by searching against the public nucleotide and protein databases.

### 4.4. Differential Gene Expression Analysis

To estimate the levels of gene expression, the clean reads from each sample were assembled into Trinity assemblies using RSEM. The expression levels, or abundances, of unigenes were then calculated using the fragments per kilobase million (FPKM) method [49]. DEG analysis of the gonad was conducted using DESeq [50]. DESeq offers statistical algorithms to detect differential expression in digital gene expression data using a model that relies on the negative binomial distribution. To control the false discovery rate, the *p*-values obtained were adjusted using the Benjamini and Hochberg approach. Genes exhibiting an adjusted *p*-value < 0.05, as calculated using DESeq, were assigned as DEGs. GO enrichment analysis of DEGs was performed using GOseq [51]. The enrichment of DEGs in KEGG pathways was analyzed using KOBAS software (version v2.0.12) [52,53].

### 4.5. Synthesis of dsRNA and RNA Interference

The Fcp3C cDNA gene fragments were obtained by amplifying restriction fragments of 366 bp using RT-PCR. These fragments were then cloned into the pGEM T-Easy vector. The Fcp3C gene, containing XbaI and HindIII restriction sites, was subsequently sub-cloned into the L4440 vector that has T7 promoters at both ends. The bacterially expressed dsFcp3C and feeding RNAi experiments were performed following a protocol reported by Lü et al. [54]. To evaluate the effect of bacterially expressed dsFcp3C on *H. parallela*, adult beetles were subjected to six replicated bioassays. In each replicate, a group of five adults was provided with 10 mm diameter elm leaf disks for feeding. Before the feeding assay, the individuals were starved for 48 h. The leaf disks were soaked in a 0.1 μg/μL dsFcp3C, dsGFP solution and ddH_2_O for 60 sec at room temperature, and then air-dried for 60 min. Based on our preliminary experiments, the leaf disks were replaced every 48 h by fresh elm leaves coated with 0.1 μg/μL dsRNA, and the feeding continued for 11 d. The survival of adult beetles was recorded. To assess the effectiveness of RNAi-mediated knockdown of target genes, an additional group of adults was reared. After feeding on dsRNA, dsGFP solution or ddH_2_O, five beetles from each treatment were randomly selected for qPCR analysis on the 7th day.

### 4.6. Gene Expression Analysis

Gene expression profiles were analyzed using quantitative real-time PCR (qPCR). The qPCR reaction was conducted on the StepOnePlus Real-time PCR system (Applied Biosystems, Foster City, CA, USA) with the SYBR Premix Ex Taq II (TaKaRa, Tokyo, Japan). Primer pairs were designed based on the nucleotide sequences using the Primer3 software (version 4.0.0, http://bioinfo.ut.ee/primer3-0.4.0/primer3/ (accessed on 8 August 2022)). The primer sequences are shown in Table S1. To ensure accurate normalization, the housekeeping gene glyceraldehyde 3-phosphate dehydrogenase was used as an internal control. Each amplification reaction consisted of a 20μL reaction mixture and followed the following conditions: initial denaturation at 95 °C for 30 s, followed by 40 cycles of denaturation at 95 °C for 5 s and annealing at 60 °C for 30 s. Relative expression levels across the samples were determined using the 2^−ΔΔCT^ method [55].

### 4.7. Microscopic Observation

Samples for examining the ovarian structure were prepared on the 11th day by dissecting the ovaries of adult females. The ovaries of females treated with ddH_2_O and dsGFP were examined using the same method as the dsFcp3C treatment. Photos were taken with a VHX-7000 digital microscope (KEYENCE, Mechelen, Belgium).

### 4.8. Statistical Analysis

Data were analyzed using Microsoft Excel, and statistical analyses were conducted using Duncan’s multiple range test in the Data Processing System software (version 7.05). All figures were processed in Adobe Illustrator. A heat map was constructed using the online mapping tool imageGP (http://www.bic.ac.cn/BIC/#/ (accessed on 15 June 2023)) [56].

## 5. Conclusions

We evaluated the effects of three host plants on the oviposition of *H. parallela*. We also studied DEGs that may be related to oviposition and that have the potential to be used as pest control targets. RNAi experiments were conducted to explore the role of Fcp3C, a crucial gene in *H. parallela*. The results enhance our understanding of the molecular mechanisms of oviposition and describe a method of identifying genes that may be useful for pest control.

## Figures and Tables

**Table 1:** Adult longevity, survival rate, fecundity and oviposition duration of *Holotrichia parallela* reared on different diet treatments.

Parameter	Mean Number of Eggs Laid Per Female	Oviposition Duration (d)	Adult Longevity (d)	Survival Rate (%)
Elm	67.4 ± 0.240 ^a^	59.3 ± 2.027 ^a^	66 ± 0.000 ^a^	90.0 ± 5.773 ^a^
Willow	21.1 ± 0.623 ^b^	46.7 ± 1.333 ^b^	66 ± 0.000 ^a^	70.0 ± 5.773 ^b^
Purpus privet	8.6 ± 0.607 ^c^	39.3 ± 4.667 ^c^	66 ± 0.000 ^a^	33.3 ± 3.333 ^c^

The lowercase letters within the column indicate significant differences (*p* < 0.05) by Duncan’s multiple range test.

## Data Availability

All raw RNA sequencing data have been deposited in the Genome Sequence Archive [57] at the National Genomics Data Center [58] (China National Center for Bioinformation/Beijing Institute of Genomics, Chinese Academy of Sciences) (GSA: CRA011589) that are publicly accessible at https://ngdc.cncb.ac.cn/gsa (accessed on 28 June 2023).

## Supplementary Materials

The following supporting information can be downloaded at: https://www.mdpi.com/article/10.3390/ijms241713138/s1.
