# Supplementary material for: Identification of Potential Gene Targets for Suppressing Oviposition in Holotrichia parallela Using Comparative Transcriptome Analysis
Source: Int J Mol Sci. 2023 Aug 24;24(17):13138. doi: 10.3390/ijms241713138 (PMC10487570; doi:10.3390/ijms241713138)

International Journal of *Molecular Sciences*

Article

Identification of Potential Gene Targets for Suppressing Oviposition in *Holotrichia parallela* through Comparative Transcriptome Analysis

Zhongjun Gong[1], Jing Zhang[1], Yanmin Li[2], Huiling Li[1], Ziqi Zhang[3], Yifan Qin[1], Yueli Jiang[1],Yun Duan[1], Tong Li[1],Jin Miao[1] and Yuqing Wu[1]*

[1] Institute of Plant Protection, Henan Academy of Agricultural Sciences, Key Laboratory of Crop Pest Control of Henan Province, Key Laboratory of Crop Integrated Pest Management of the Southern of North China, Ministry of Agriculture of the People's Republic of China, Zhengzhou 450002, China; gongzj_2@hotmail.com (Z.G.)
[2] Institute of Horticulture, Henan Academy of Agricultural Sciences, Zhengzhou 450002, China
[3] Institute of Plant Protection, Luoyang Academy of Agricultural and Forestry Sciences, Luoyang 471027, China
* Correspondence: yuqingwu36@hotmail.com

Citation: Gong, Z.; Zhang, J.; Li, Y.; Li, H.; Zhang, Z.; Qin, Y.; Jiang, Y.; Duan, Y.; Li, T.; Miao, J.; et al. Identification of Potential Gene Targets for Suppressing Oviposition in *Holotrichia parallela* Using Comparative Transcriptome Analysis. *Int. J. Mol. Sci.* **2023**,*24*, 13138. https://doi.org/10.3390/ijms241713138

Academic Editors: Elena N. Elpidina and Yakov E. Dunaevsky

Received: 7 July 2023
Revised: 18 August 2023
Accepted: 22 August 2023
Published: 23 August 2023

Copyright: © 2023 by the authors. Submitted for possible open access publication under the terms and conditions of the Creative Commons Attribution (CC BY) license (https://creativecommons.org/licenses/by/4.0/).

Supplementary Figures

Figure S1. Species classification.

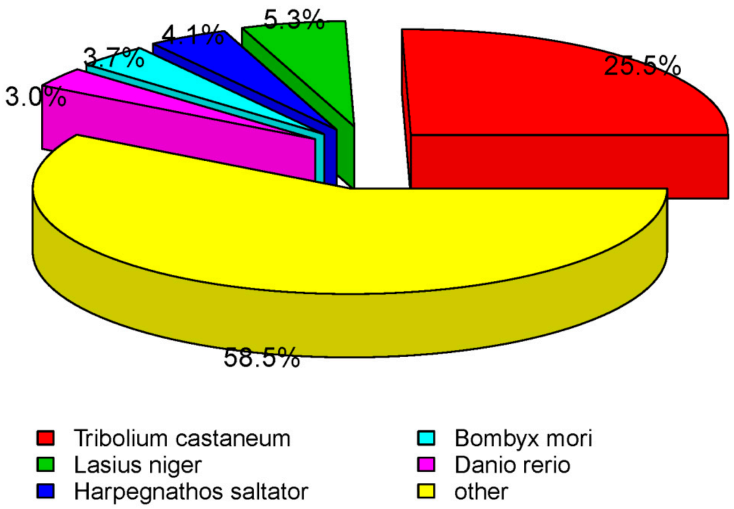

Figure S2. KOG Function Classification.

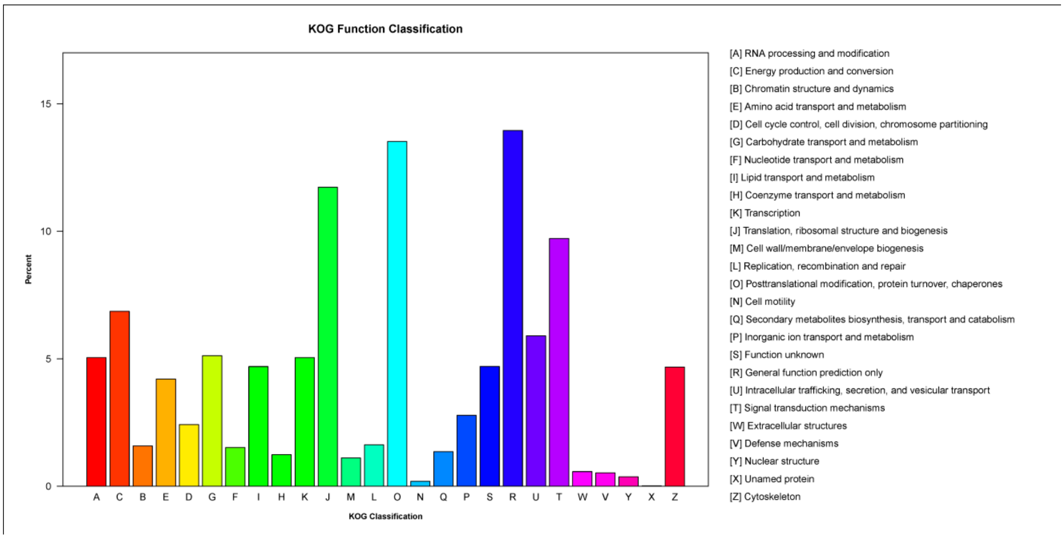

Supplement: Supplementary file 1 [file ijms-24-13138-s001.zip › Supplementary_Material_Figures.pdf]
